# Supplementary material for: Structural features within precursor microRNA-20a regulate Dicer-TRBP processing
Source: bioRxiv. 2025 May 11:2025.05.07.652689. Preprint. [Version 1] doi: 10.1101/2025.05.07.652689 (PMC12247985; doi:10.1101/2025.05.07.652689)
Supplement: Supplement 1 [file media-1.pdf]

SUPPORTING INFORMATION

Structural features within precursor microRNA-20a regulate Dicer-TRBP processing

Yaping Liu[1†], Cade T. Harkner[2†], Megan N. Westwood[1], Aldrex Munsayac[2], and Sarah C. Keane[1,2*]

[1]Biophysics Program, University of Michigan, 930 N. University Avenue, Ann Arbor, MI 48109, USA

[2]Department of Chemistry, University of Michigan, 930 N. University Avenue, Ann Arbor, MI 48109, USA

[†]These authors contributed equally

[*]Author to whom correspondence should be addressed, sckeane@umich.edu

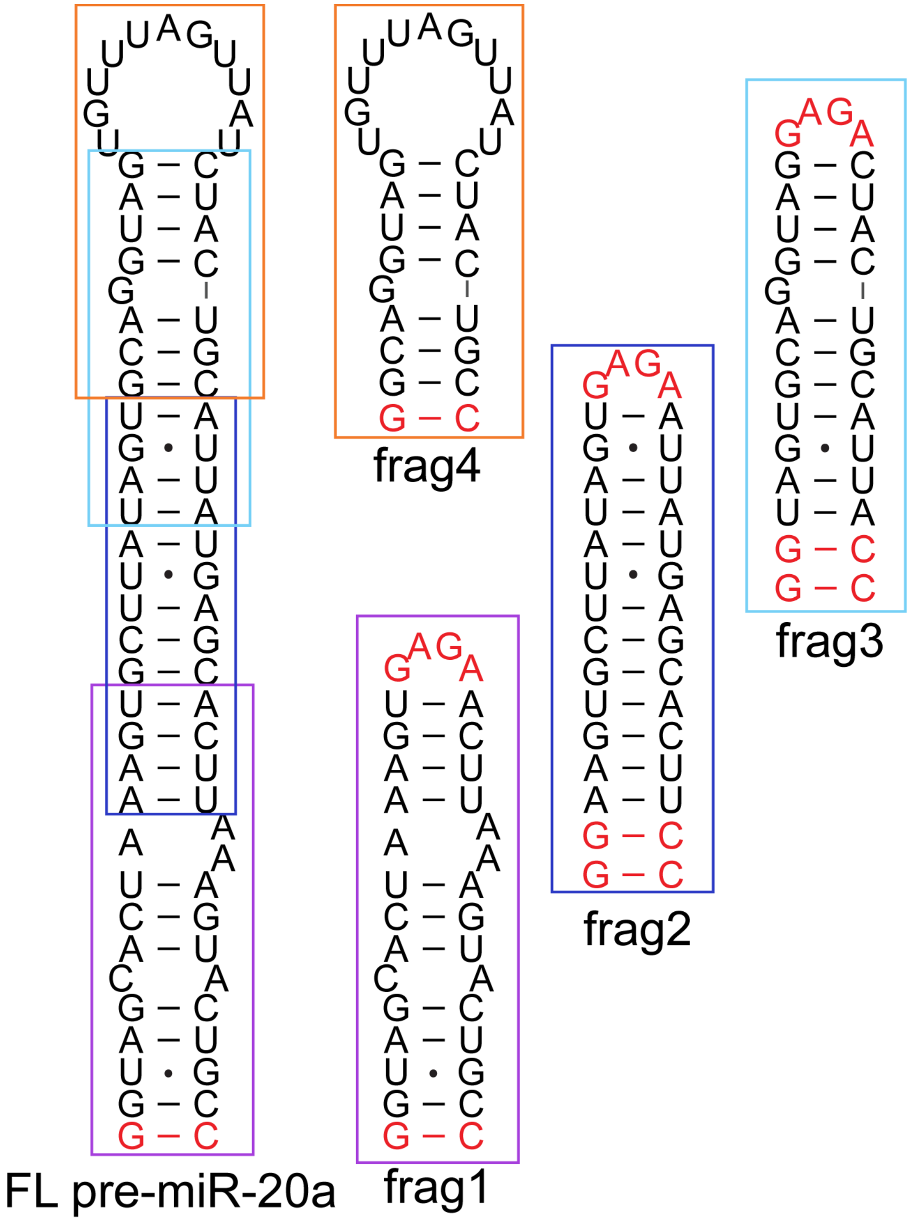

Figure S1. Oligonucleotide controls for NMR chemical shift assignment of FL pre-miR-20a. Four oligonucleotide controls were designed to cover the entire FL pre-miR-20a sequence. Non-native tetraloops (red) were included to cap oligos that truncated the apical loop.

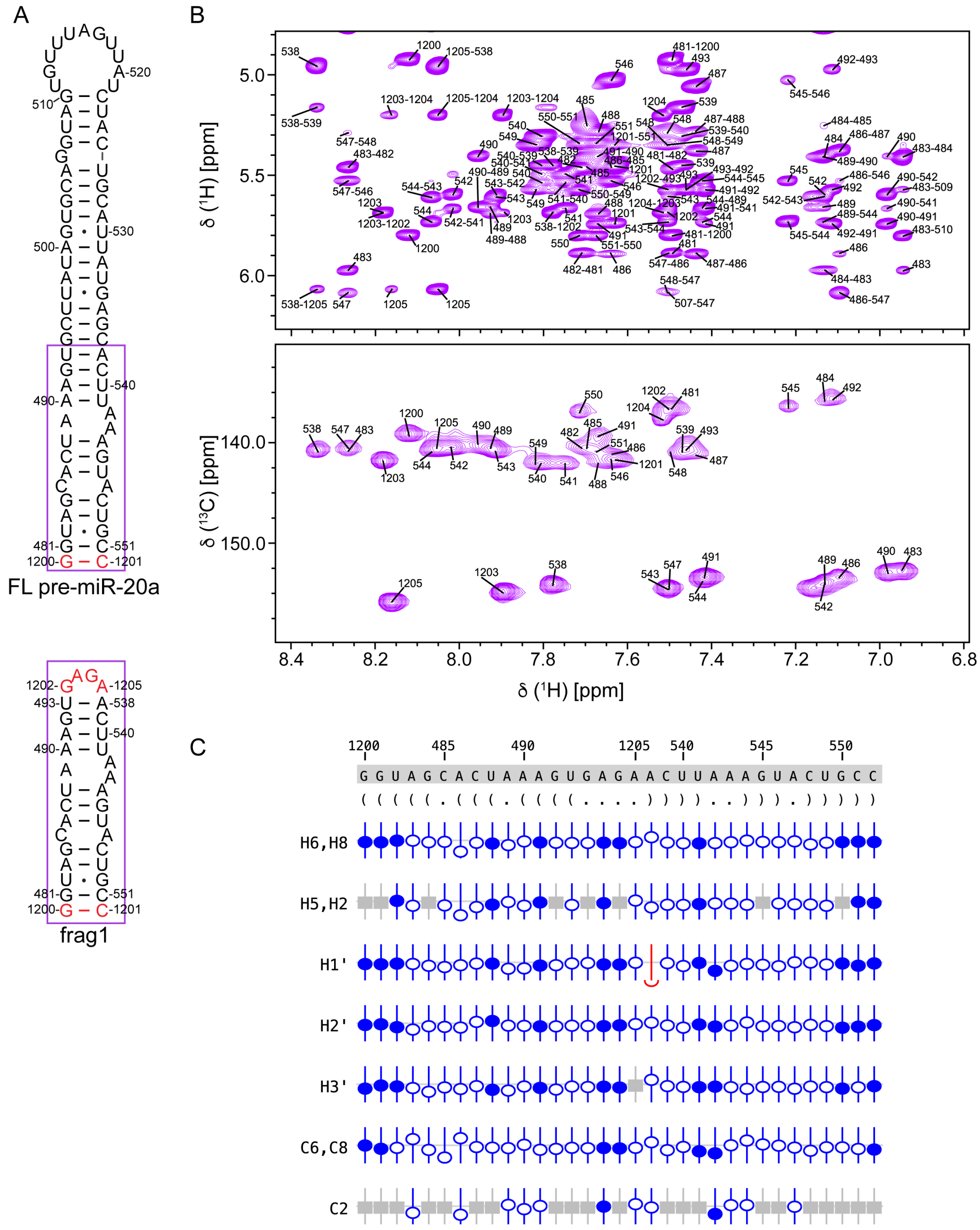

Figure S2. **Assigned chemical shifts of frag1.** (A) Secondary structure of FL pre-miR-20a and 20a-frag1. (B) ^{1}H-^{1}H NOESY (top) and ^{1}H-^{13}C HMQC (bottom) of 20a-frag1. (C) Sequence analysis and validation of 20a-frag1 chemical shift assignments. Nucleotide numbering, sequence, and secondary structure in Vienna format for 20a-frag1 RNA. NMRViewJ chemical shift prediction software was used to validate proton (H6/H8, H5/H2, H1′, H2′, H3′) and carbon (C6/C8, C2) assignments. Assigned atoms are represented with blue circles (open and closed), while grey boxes denote atoms that are not present in a given base. Deviation from the predicted chemical shift is shown with the offset from the center. Filled circles indicate that there are chemical shifts for atoms with the same set of attributes in the BMRB. Open circles indicate atoms that have a prediction, but for which no exact matches of the attributes are available in the BMRB.

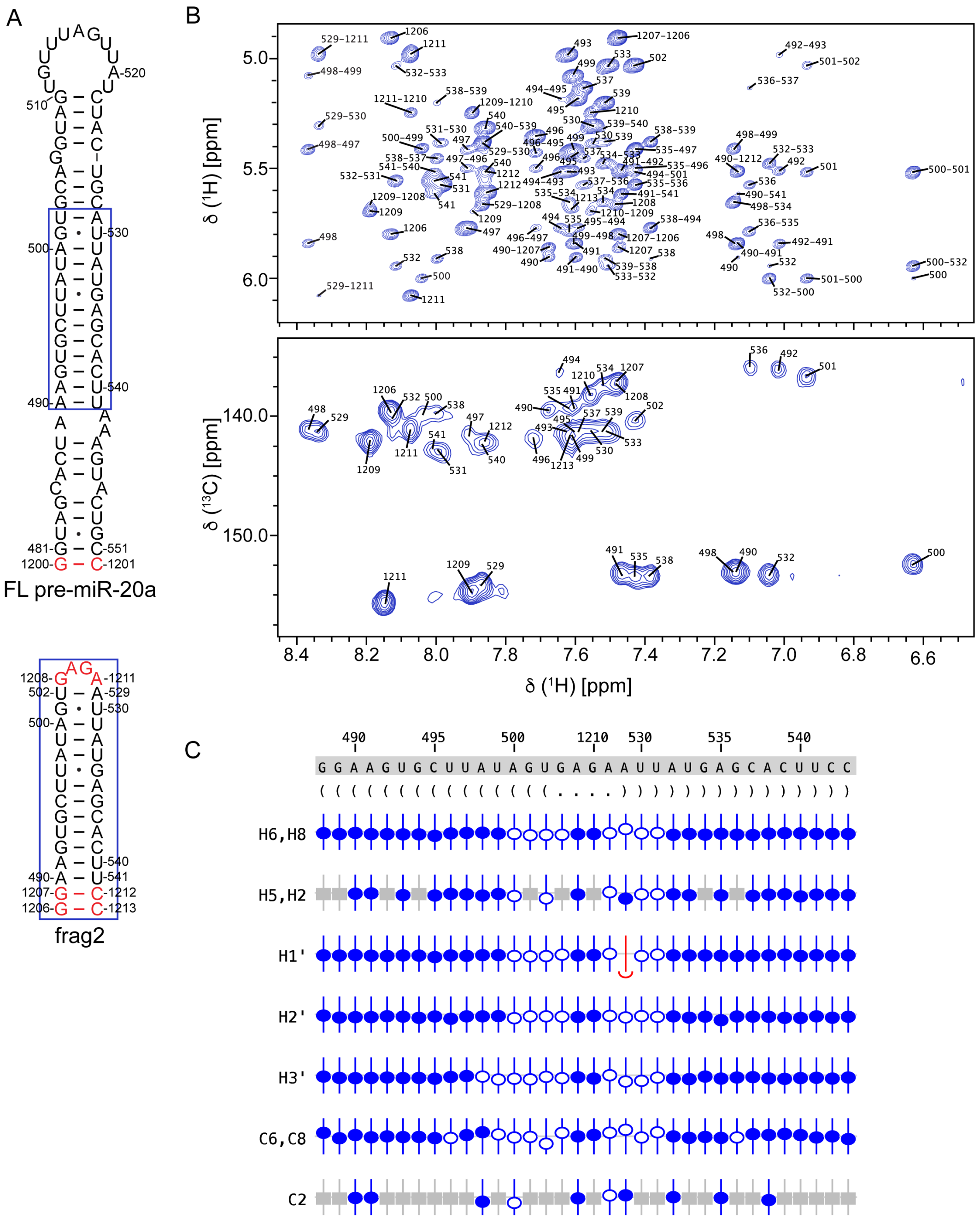

Figure S3. **Assigned chemical shifts of frag2.** (A) Secondary structure of FL pre-miR-20a and 20a-frag2. (B) ^{1}H-^{1}H NOESY (top) and ^{1}H-^{13}C HMQC (bottom) of 20a-frag2. (C) Sequence analysis and validation of 20a-frag2 chemical shift assignments. Nucleotide numbering, sequence, and secondary structure in Vienna format for 20a-frag2 RNA. NMRViewJ chemical shift prediction software was used to validate proton (H6/H8, H5/H2, H1′, H2′, H3′) and carbon (C6/C8, C2) assignments. Assigned atoms are represented with blue circles (open and closed), while grey boxes denote atoms that are not present in a given base. Deviation from the predicted chemical shift is shown with the offset from the center. Filled circles indicate that there are chemical shifts for atoms with the same set of attributes in the BMRB. Open circles indicate atoms that have a prediction, but for which no exact matches of the attributes are available in the BMRB.

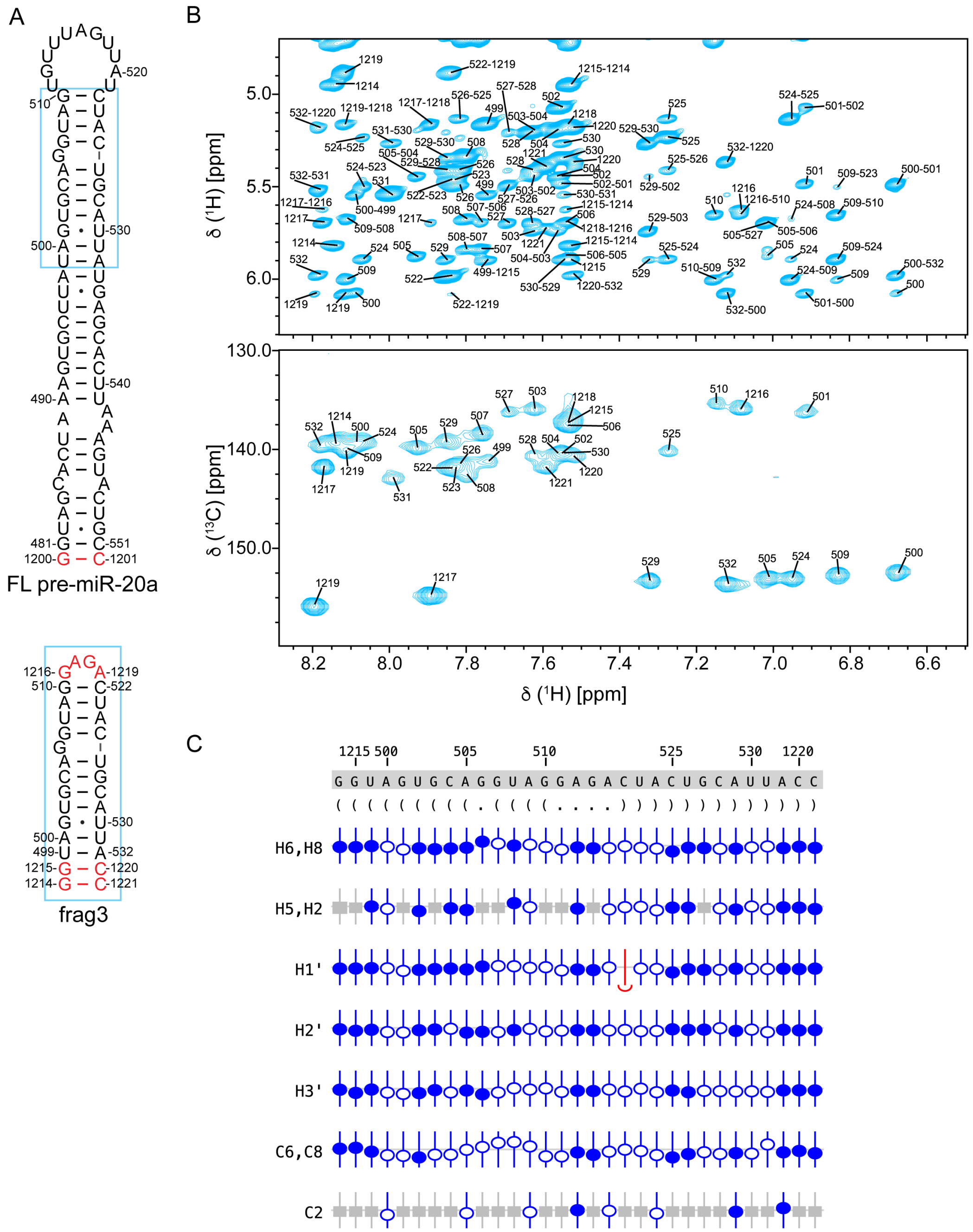

Figure S4. **Assigned chemical shifts of frag3.** (A) Secondary structure of FL pre-miR-20a and 20a-frag3. (B) ^{1}H-^{1}H NOESY (top) and ^{1}H-^{13}C HMQC (bottom) of 20a-frag3. (C) Sequence analysis and validation of 20a-frag3 chemical shift assignments. Nucleotide numbering, sequence, and secondary structure in Vienna format for 20a-frag3 RNA. NMRViewJ chemical shift prediction software was used to validate proton (H6/H8, H5/H2, H1′, H2′, H3′) and carbon (C6/C8, C2) assignments. Assigned atoms are represented with blue circles (open and closed), while grey boxes denote atoms that are not present in a given base. Deviation from the predicted chemical shift is shown with the offset from the center. Filled circles indicate that there are chemical shifts for atoms with the same set of attributes in the BMRB. Open circles indicate atoms that have a prediction, but for which no exact matches of the attributes are available in the BMRB.

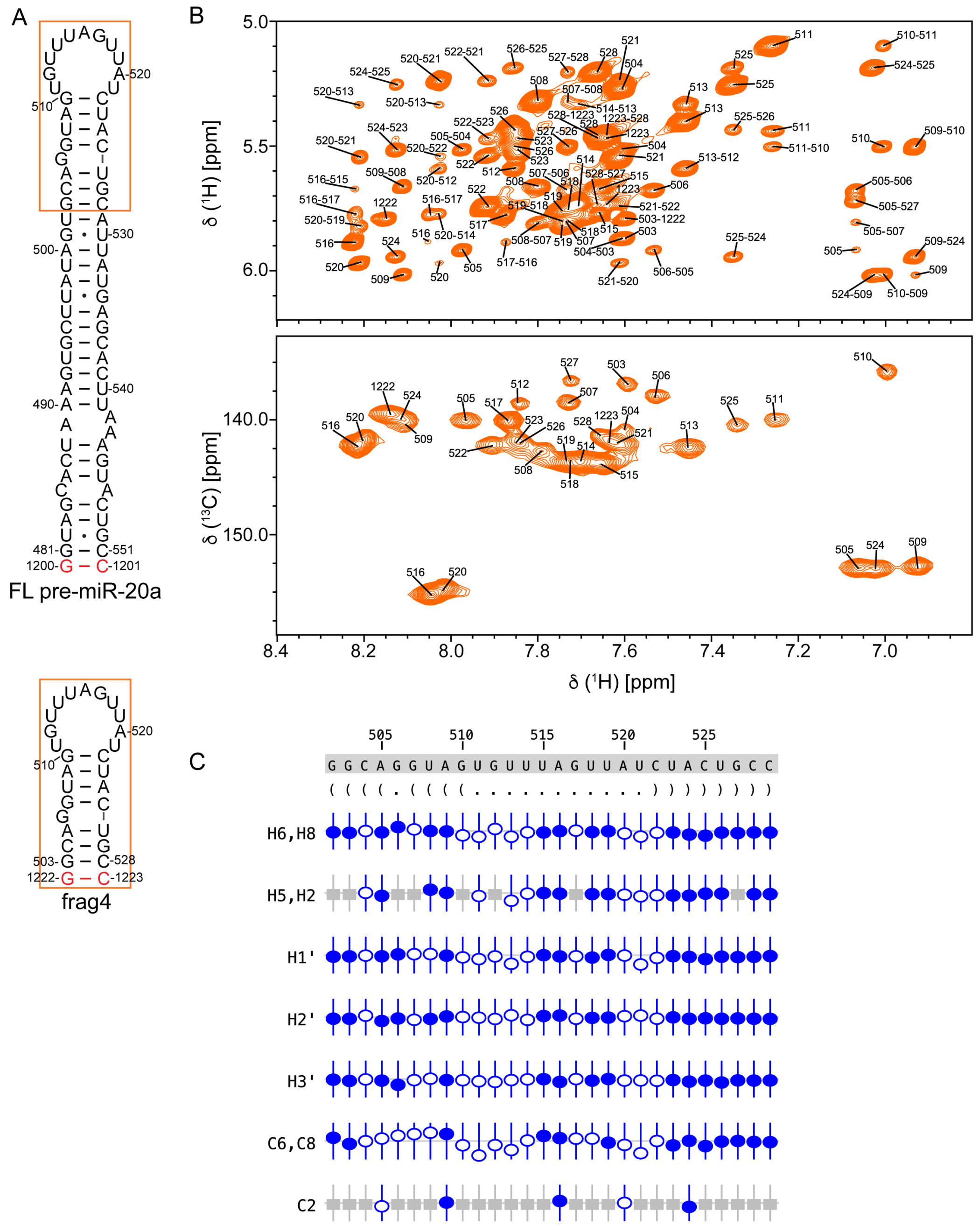

Figure S5. **Assigned chemical shifts of frag4.** (A) Secondary structure of FL pre-miR-20a and 20a-frag4. (B) ^{1}H-^{1}H NOESY (top) and ^{1}H-^{13}C HMQC (bottom) of 20a-frag4. (C) Sequence analysis and validation of 20a-frag4 chemical shift assignments. Nucleotide numbering, sequence, and secondary structure in Vienna format for 20a-frag4 RNA. NMRViewJ chemical shift prediction software was used to validate proton (H6/H8, H5/H2, H1′, H2′, H3′) and carbon (C6/C8, C2) assignments. Assigned atoms are represented with blue circles (open and closed), while grey boxes denote atoms that are not present in a given base. Deviation from the predicted chemical shift is shown with the offset from the center. Filled circles indicate that there are chemical shifts for atoms with the same set of attributes in the BMRB. Open circles indicate atoms that have a prediction, but for which no exact matches of the attributes are available in the BMRB.

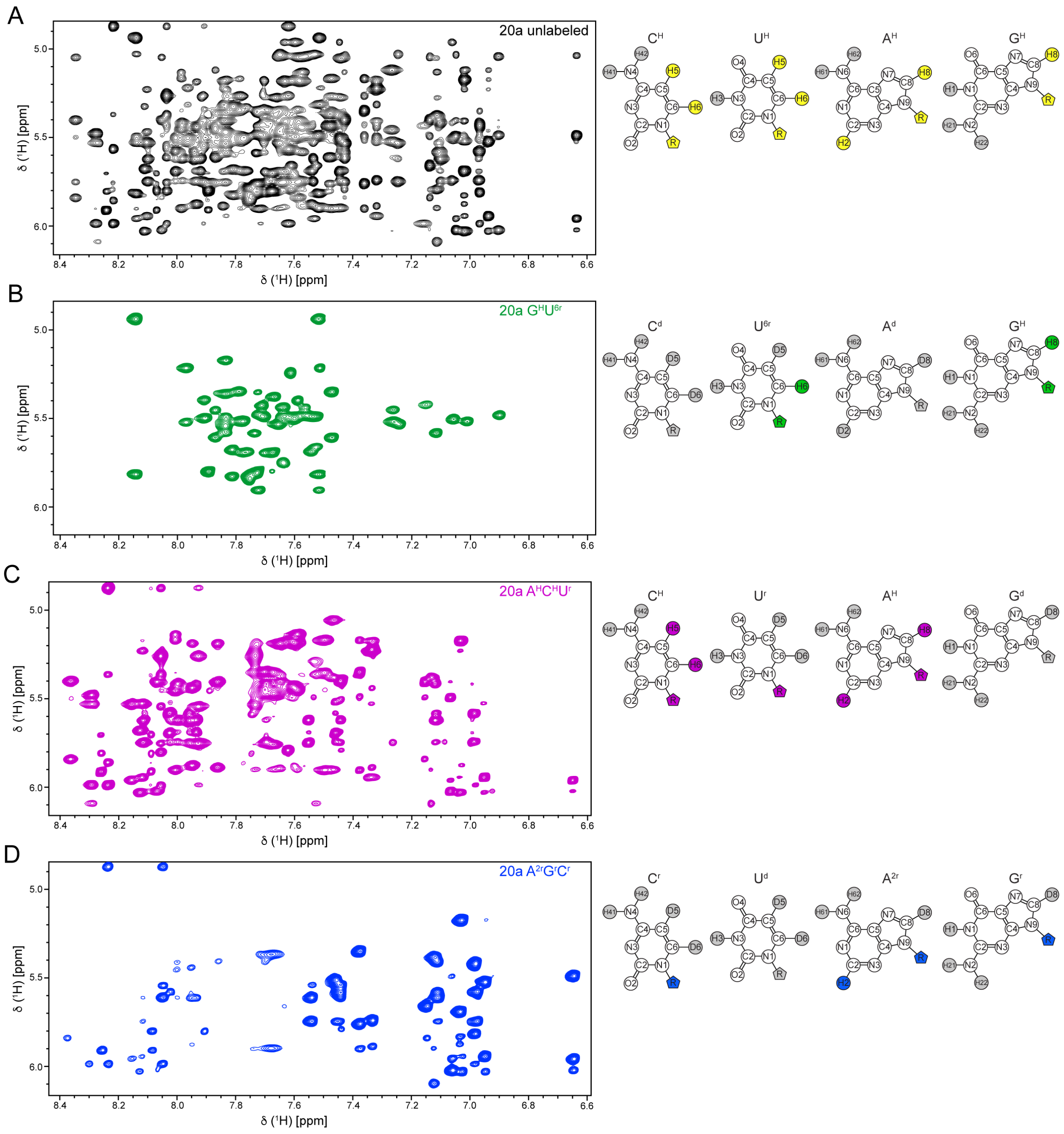

Figure S6. **Deuterium labeling improves spectral quality by reducing overlap.** ^1H-^1H NOESY spectra of (A) unlabeled (fully protiated), (B) G^HU^{6r}-labeled, (C) $A^HC^HU^r$-labeled, and (D) $A^{2r}G^rC^r$-labeled FL pre-miR-20a RNAs. Chemical structures of the four nucleosides are shown to the right of each spectrum. Sites of the selective deuteration and exchangeable protons are shaded gray while non-exchangeable protons are colored according to each spectrum.

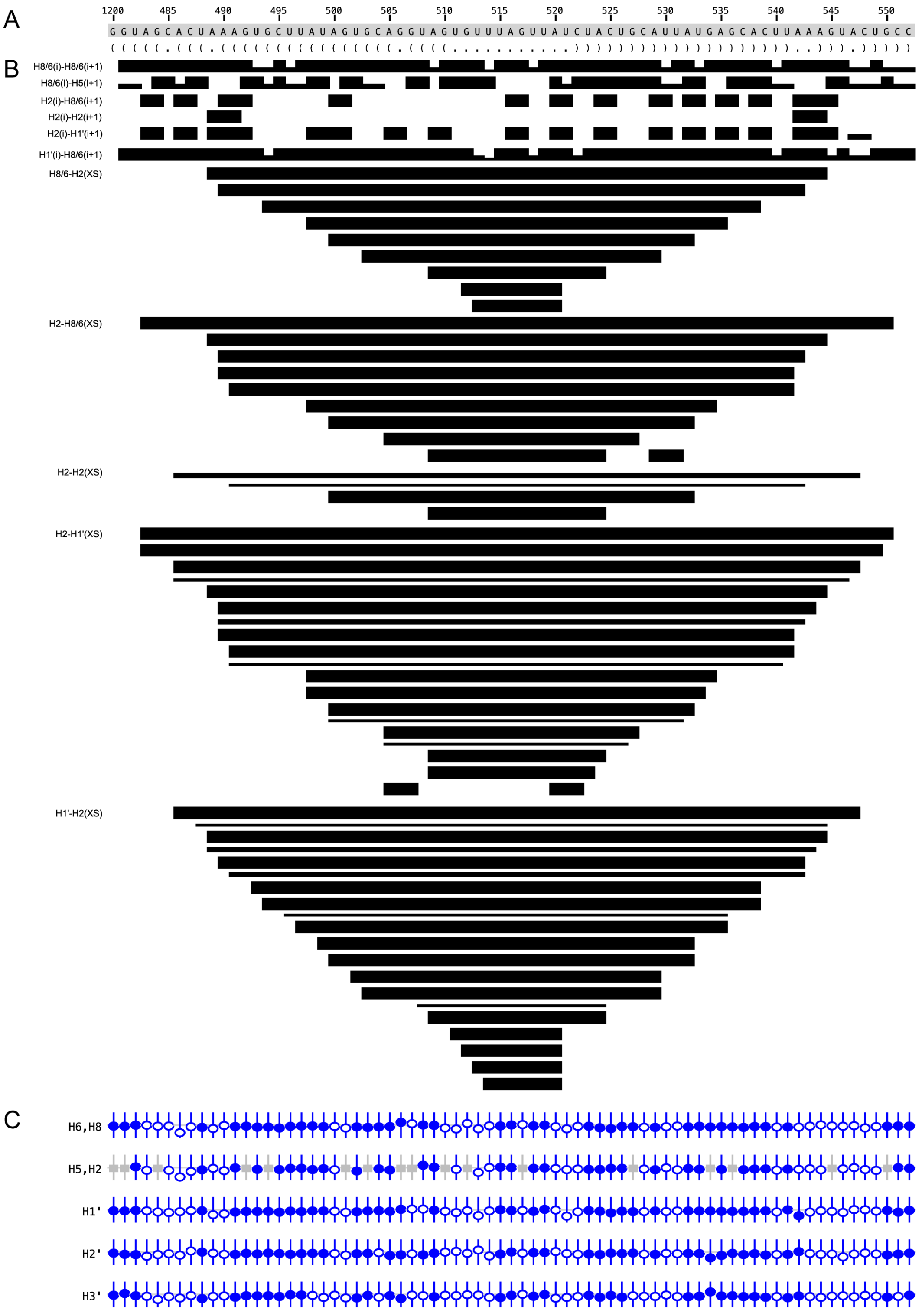

Figure S7. **Summary of the secondary structure, NOE connectivity, and chemical shift assignment validation for FL pre-miR-20a.** (A) Sequence and secondary structure of FL pre-miR-20a. (B) Inter-residue NOE upper limit restraints for specified proton pairs used in structure calculations are denoted with black bars. The thickness of the bar is representative of the strength of the measured NOE. (C) Chemical shift validation from NMRViewJ.

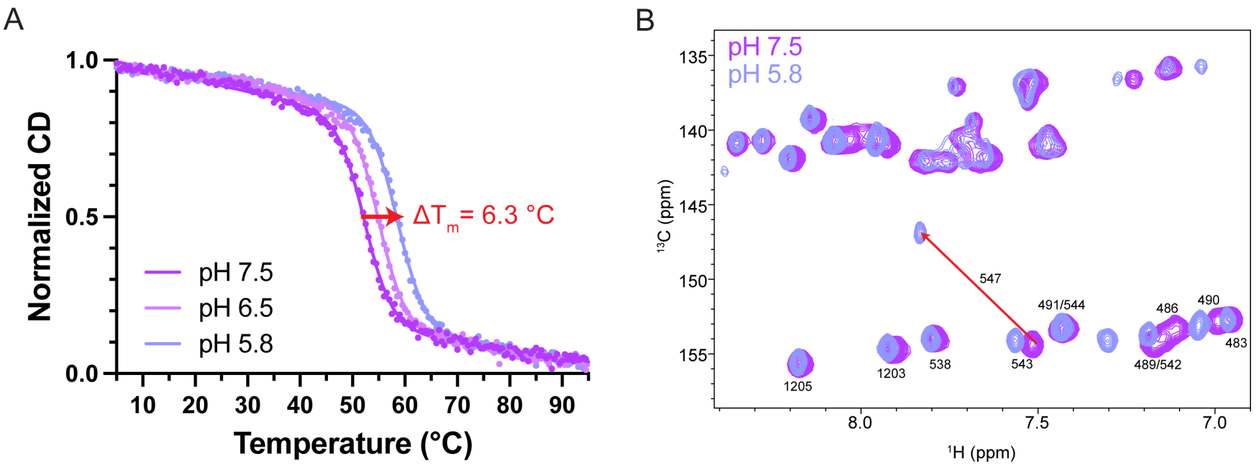

Figure S8. A547 is protonated at low pH and forms a non-canonical base pair with C485. (A) Normalized CD-thermal denaturation curves of 20a-frag1 at pH 7.5 (magenta), pH 6.5 (pink), and pH 5.8 (blue). (B) ^{1}H-^{13}C HMQC overlay of 20a-frag1 at pH 7.5 (magenta) and pH 5.8 (blue). Arrow indicates the significant shift of the A547 C2-H2 peak consistent with protonation at low pH.

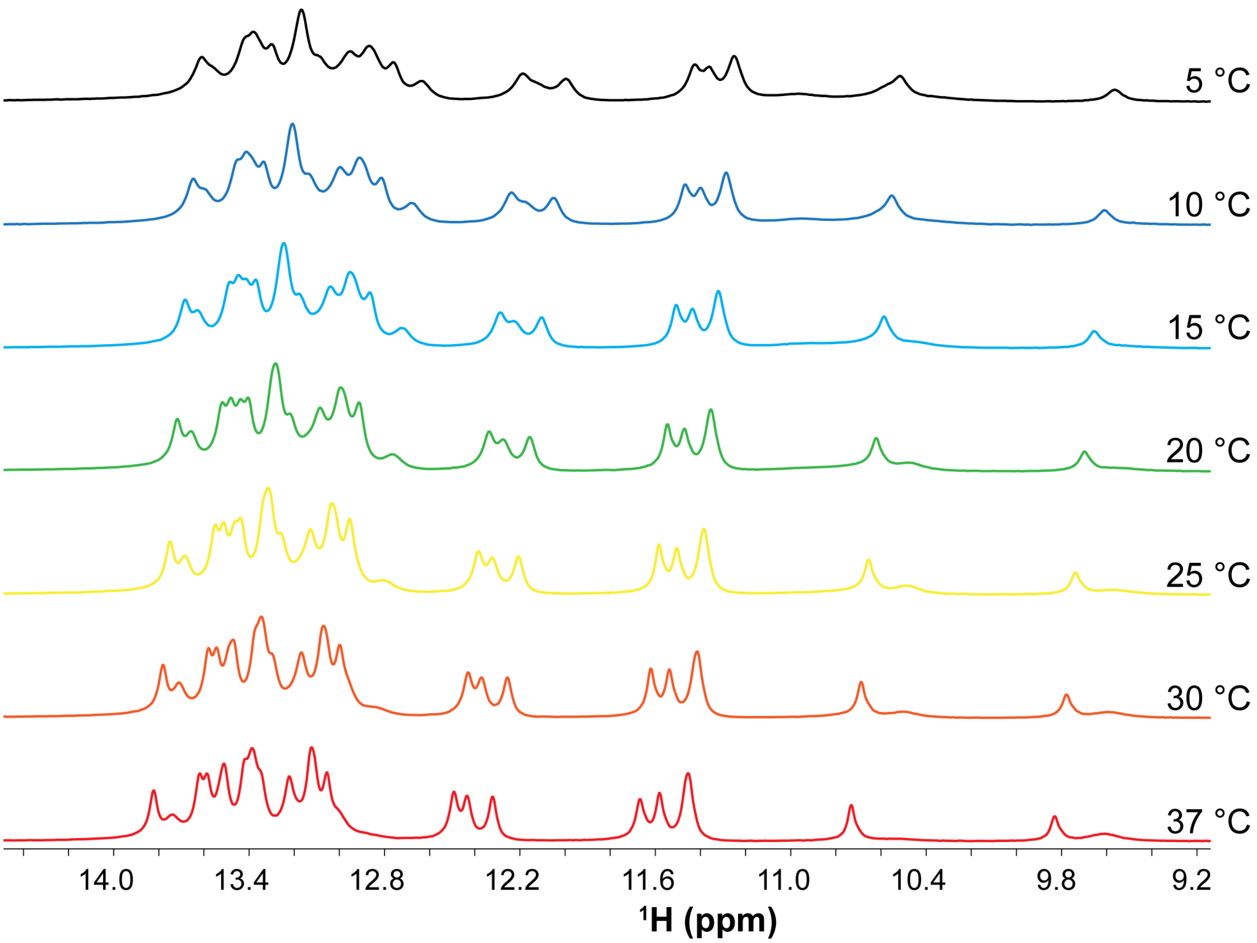

Figure S9. Imino region of ^{1}H spectra of FL pre-miR-20a as a function of temperature. The NMR spectra were recorded at 0.4 mM RNA concentration, 50 mM K-phosphate buffer, pH 6, 1 mM $MgCl_2$ and 90% H_2O/10% D_2O at 600 MHz and at temperatures between 5 °C and 37 °C.

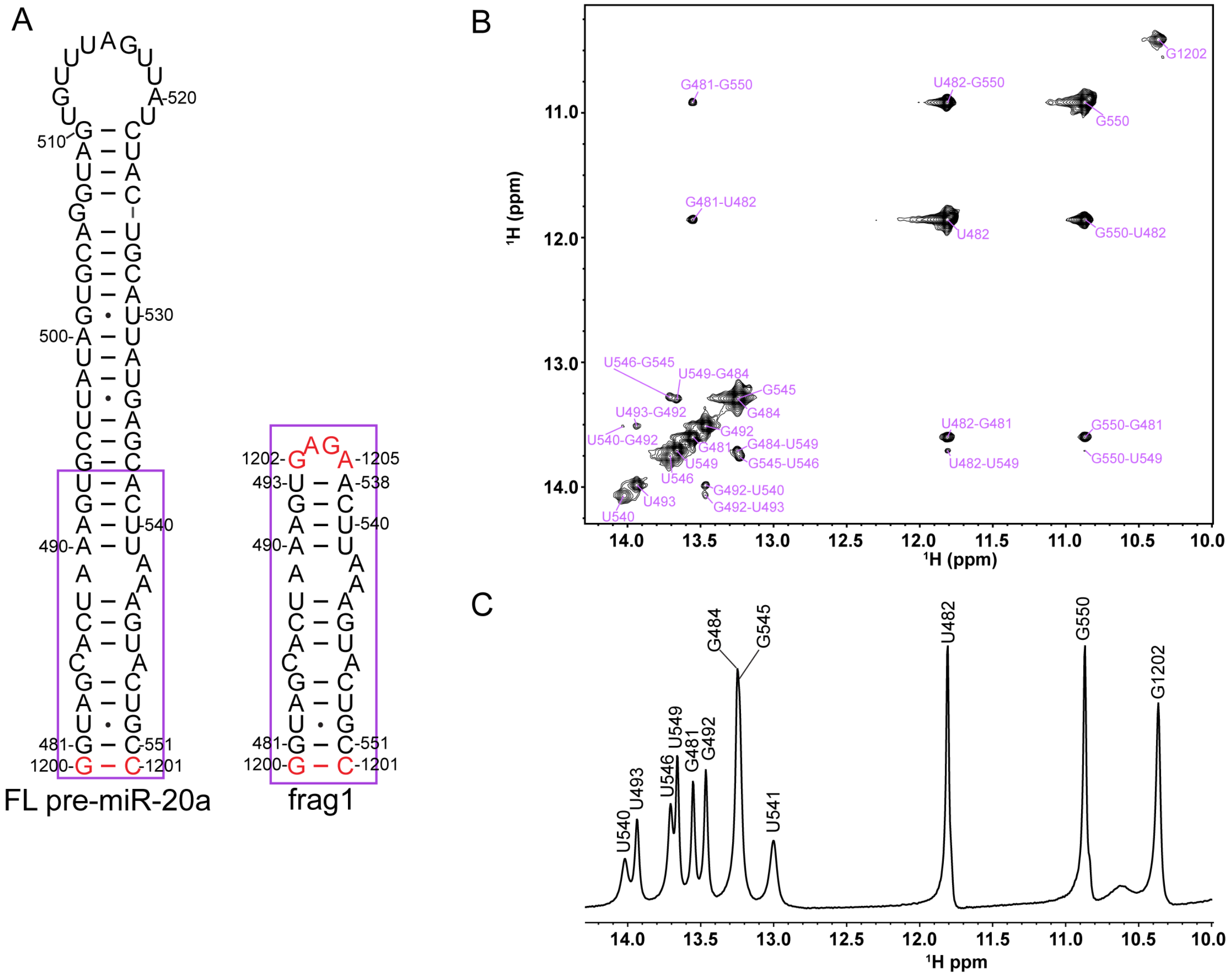

Figure S10. **Imino proton resonances of 20a-frag1.** (A) Secondary structure of FL pre-miR-20a and 20a-frag1. (B) Imino-imino region of 2D ^{1}H-^{1}H NOESY spectrum of 20a-frag1. (C) 1D ^{1}H NMR spectrum of 20a-frag1. The NMR spectra were recorded at 0.5 mM RNA concentration, 50 mM K-phosphate buffer, pH 6, 1 mM $MgCl_2$ and 90% H_2O/10% D_2O at 600 MHz and at 15 °C.

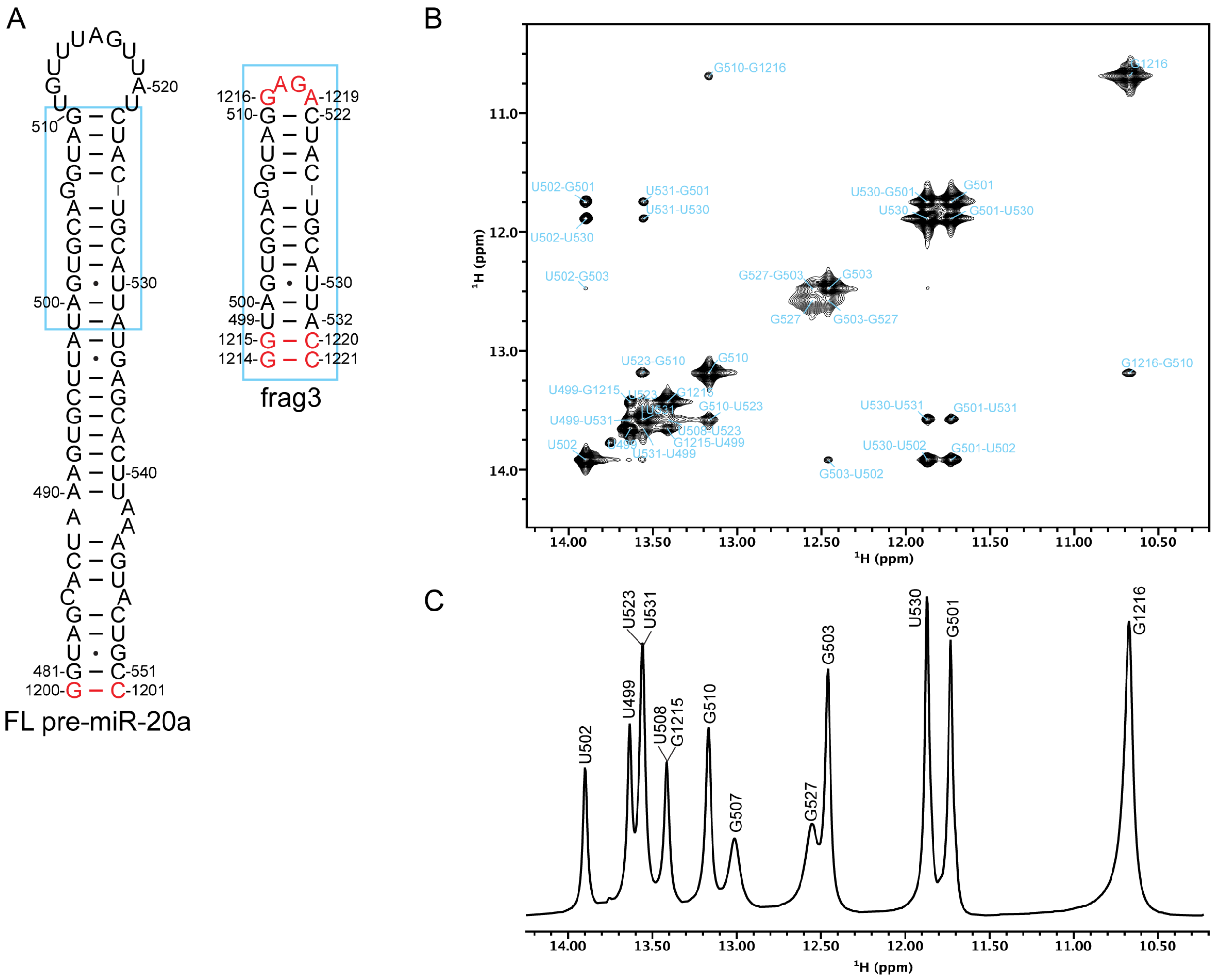

Figure S11. **Imino proton resonances of 20a-frag3.** (A) Secondary structure of FL pre-miR-20a and 20a-frag3. (B) Imino-imino region of 2D ^{1}H-^{1}H NOESY spectrum of 20a-frag3. (C) 1D ^{1}H NMR spectrum of 20a-frag3. The NMR spectra were recorded at 0.5 mM RNA concentration, 50 mM K-phosphate buffer, pH 6, 1 mM $MgCl_2$ and 90% H_2O/10% D_2O at 600 MHz and at 0 °C.

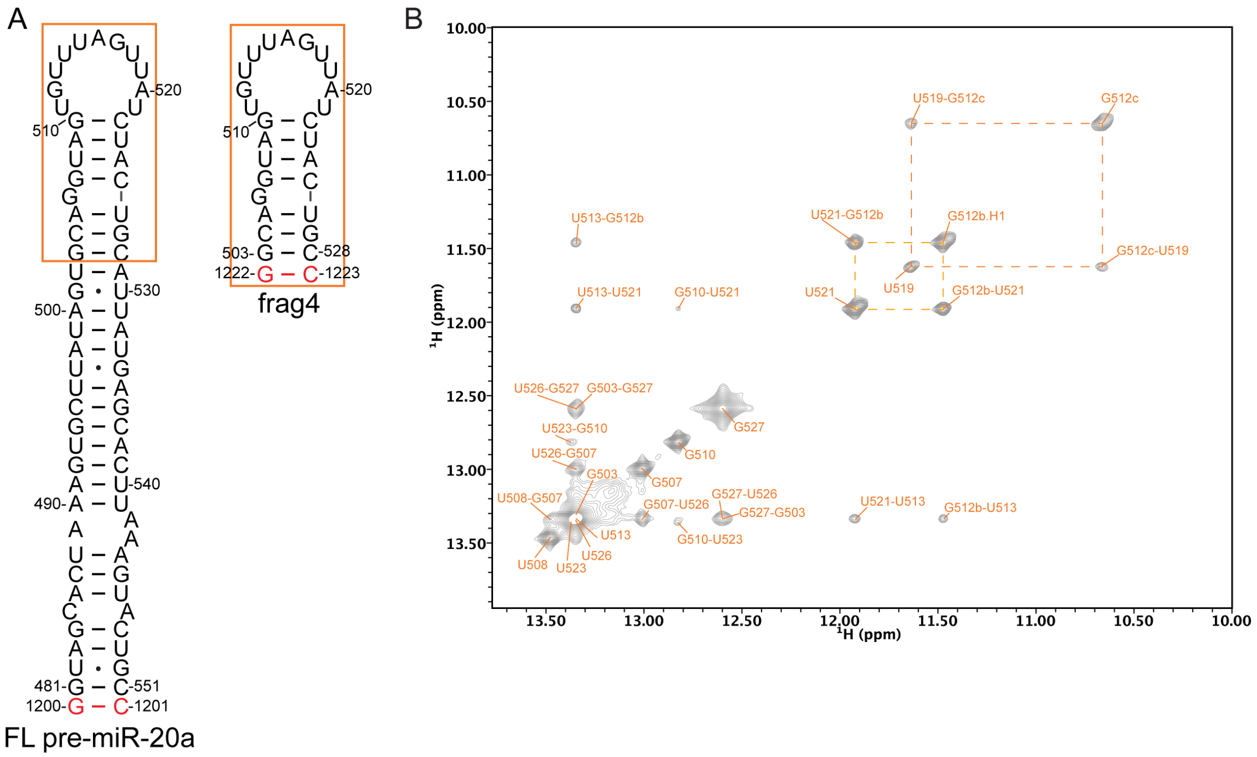

Figure S12. **Imino proton resonances of 20a-frag4.** (A) Secondary structure of FL pre-miR-20a and 20a-frag4. (B) Imino-imino region of 2D ^{1}H-^{1}H NOESY spectrum of 20a-frag4. The NMR spectra were recorded at 0.5 mM RNA concentration, 50 mM K-phosphate buffer, pH 6.5, 1 mM $MgCl_2$ and 90% H_2O/10% D_2O at 600 MHz and at 10 °C.

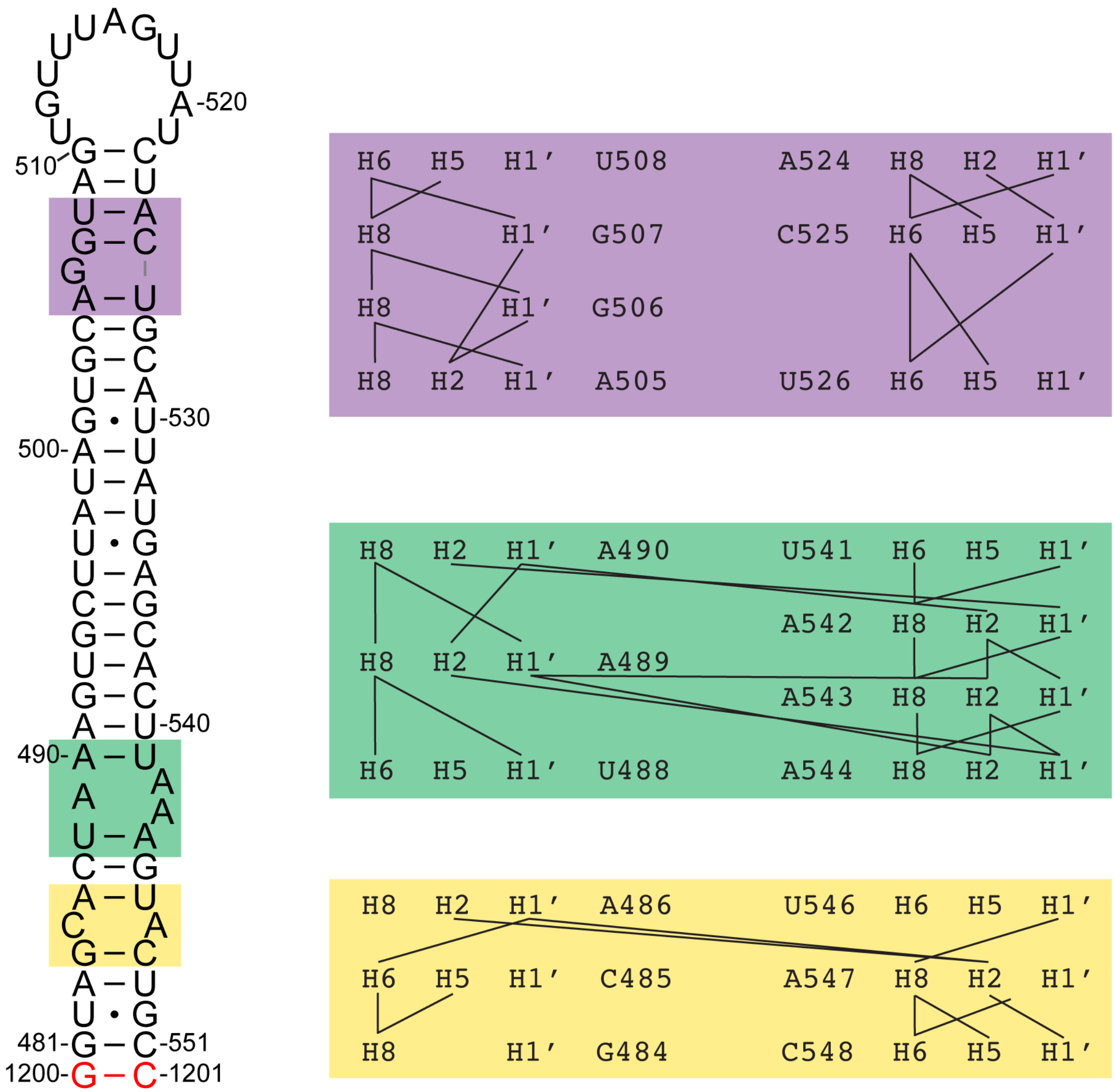

Figure S13. Summary of inter-residue NOEs for the G bulge (purple box), the A-rich 1x2 internal loop (green box), and the CA mismatch (yellow box).

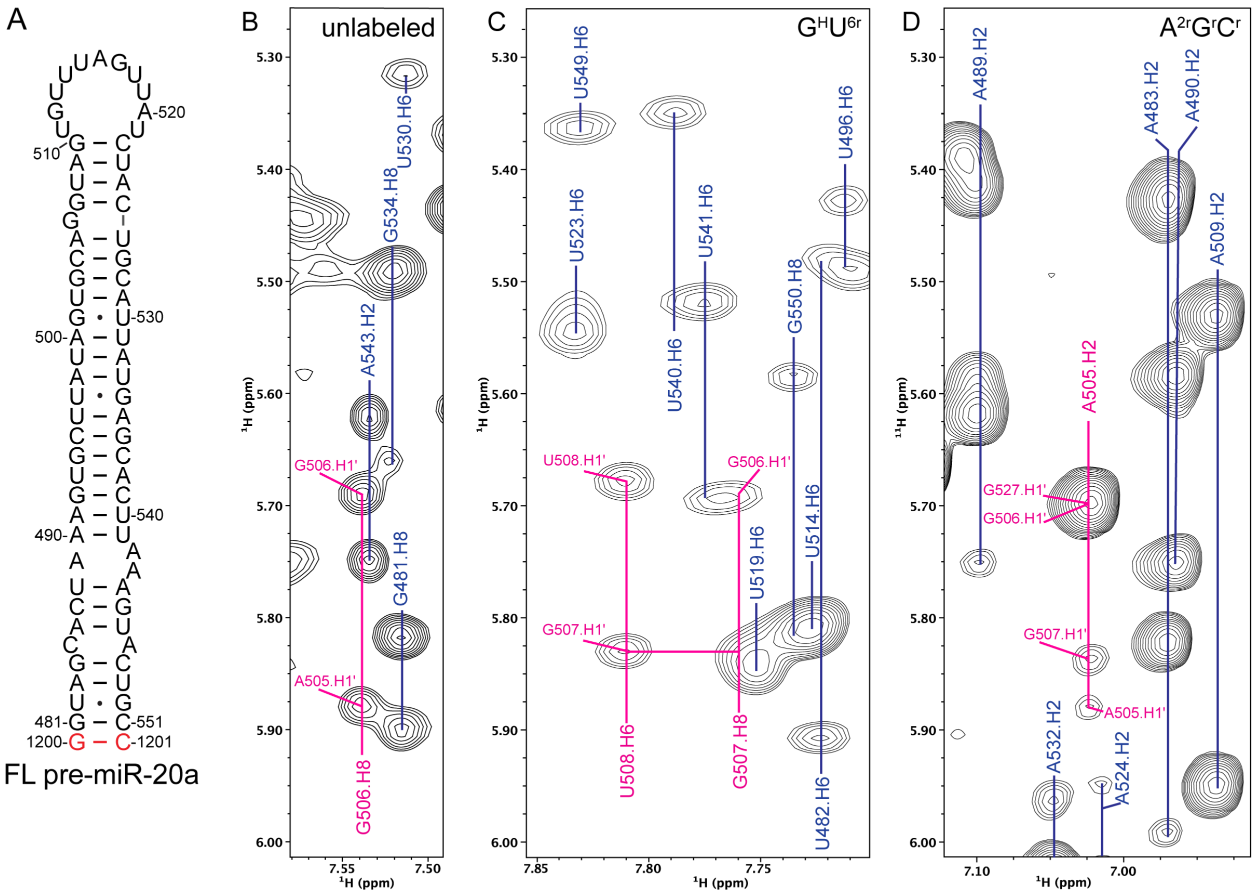

Figure S14. **NOEs of residues near the G bulge.** (A) Secondary structure of FL pre-miR-20a. (B) Region of the fully-protiated FL pre-miR-20a ^{1}H-^{1}H NOESY spectrum, highlighting NOEs with G506.H8 in pink. Other assignments noted in blue. (C) Region of the ^{1}H-^{1}H NOESY spectrum of G^{H}U^{6r} labeled FL pre-miR-20a, highlighting NOEs connecting G507.H8 and U508.H6 in pink. Other assignments noted in blue. (D) Region of the ^{1}H-^{1}H NOESY spectrum of A^{2r}G^{r}C^r labeled FL pre-miR-20a, highlighting NOEs with A505.H2 in pink. Other assignments noted in blue.

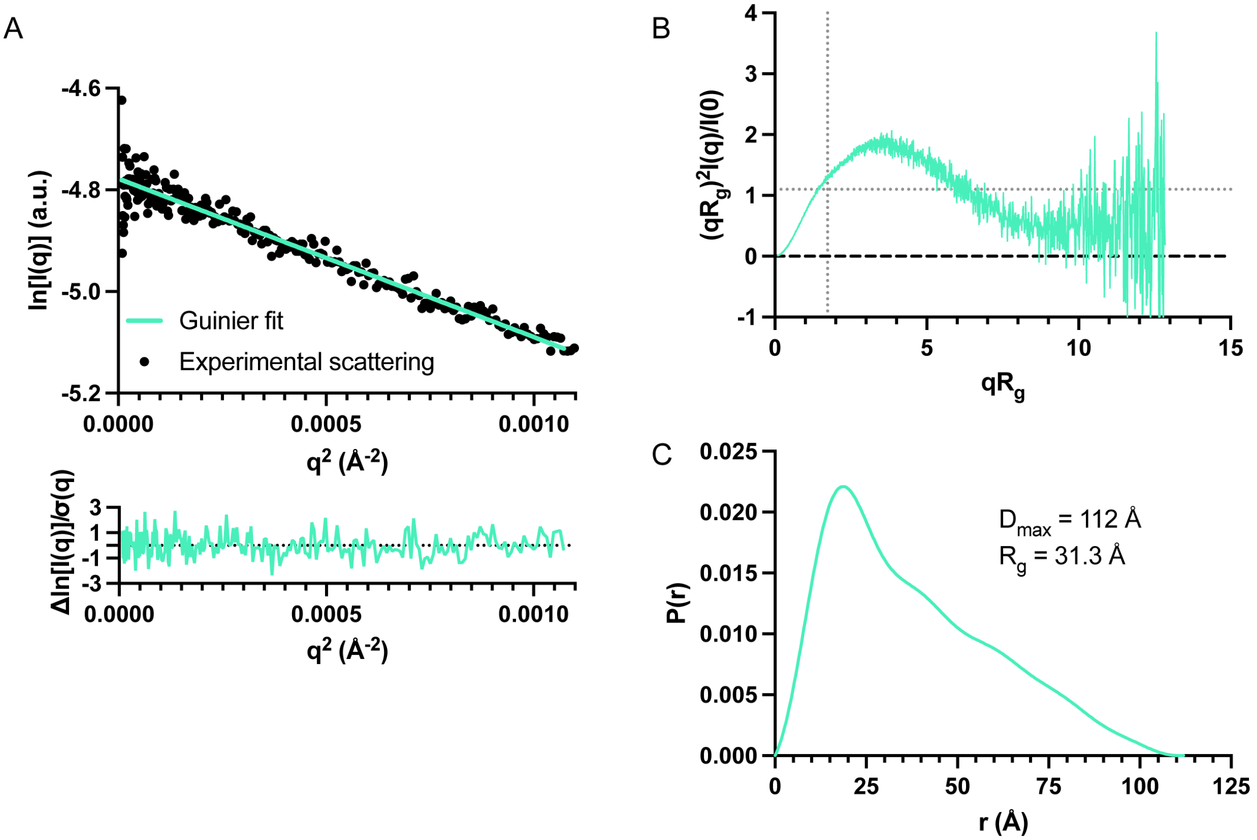

Figure S15. **Experimental SAXS data of FL pre-miR-20a.** (A) Guinier analysis of FL pre-miR-20a (top) with normalized residuals (bottom). (B) Dimensionless Kratky plot of FL pre-miR-20a. The dashed gray lines on the plot are guidelines for a peak position of a perfectly globular system. (C) Normalized pair distance distribution function of the FL pre-miR-20a.

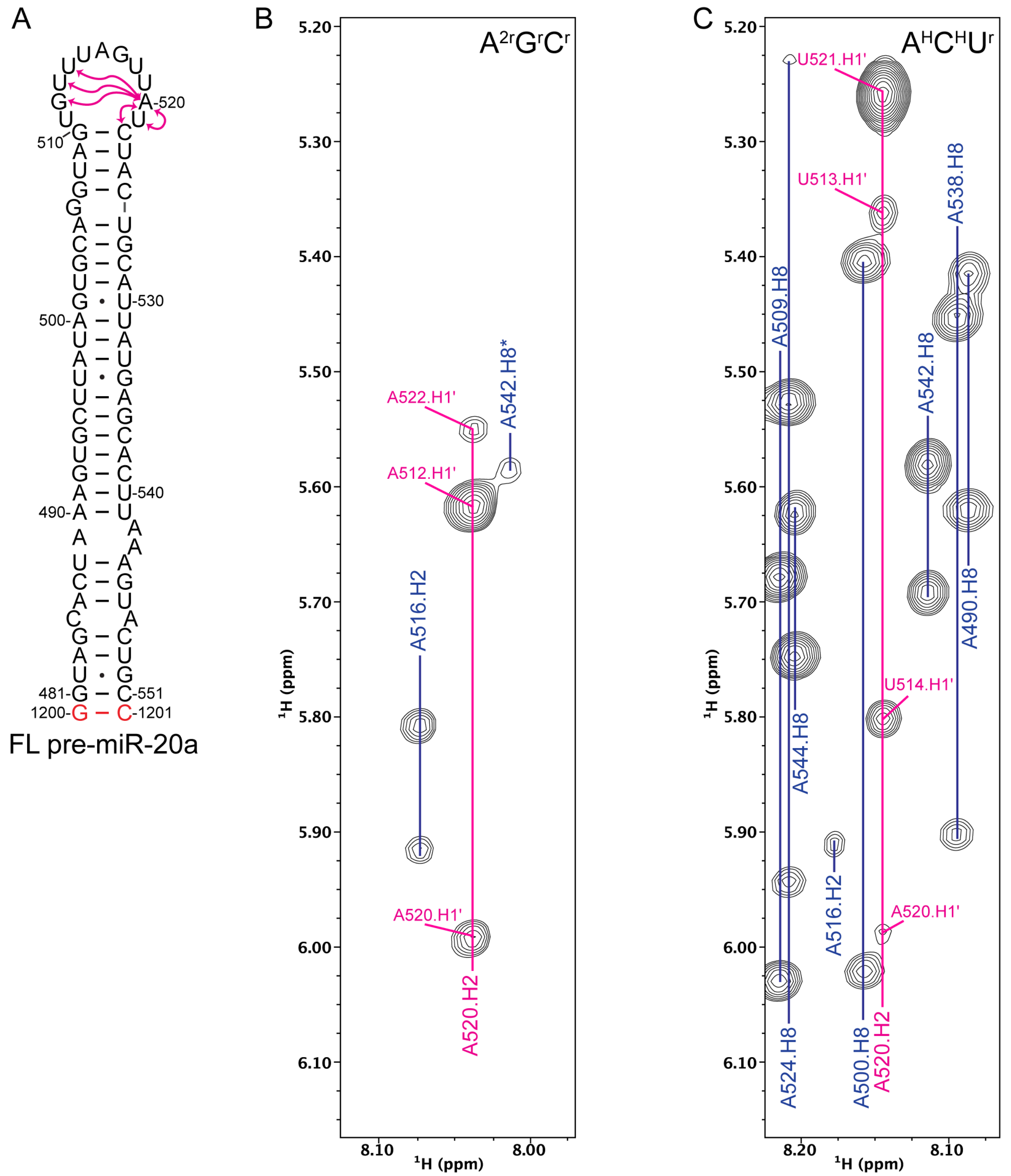

Figure S16. Extensive cross-strand NOE network in the apical loop of FL pre-miR-20a. (A) Secondary structure of FL pre-miR-20a highlighting extensive NOE connectivities (pink arrows). (B) Portion of 2D ^{1}H-^{1}H NOESY spectrum of A^{2r}G^{r}C^{r}-labeled FL pre-miR-20a. NOEs with A520.H2 are labeled in pink. Other assignments are noted in blue. The * on A542.H8 indicates a breakthrough signal due to incomplete deuteration at the adenosine C8 position. (C) Portion of 2D ^{1}H-^{1}H NOESY spectrum of A^{H}C^{H}U^{r}-labeled FL pre-miR-20a. NOEs with A520.H2 are labeled in pink. Other assignments are noted in blue.

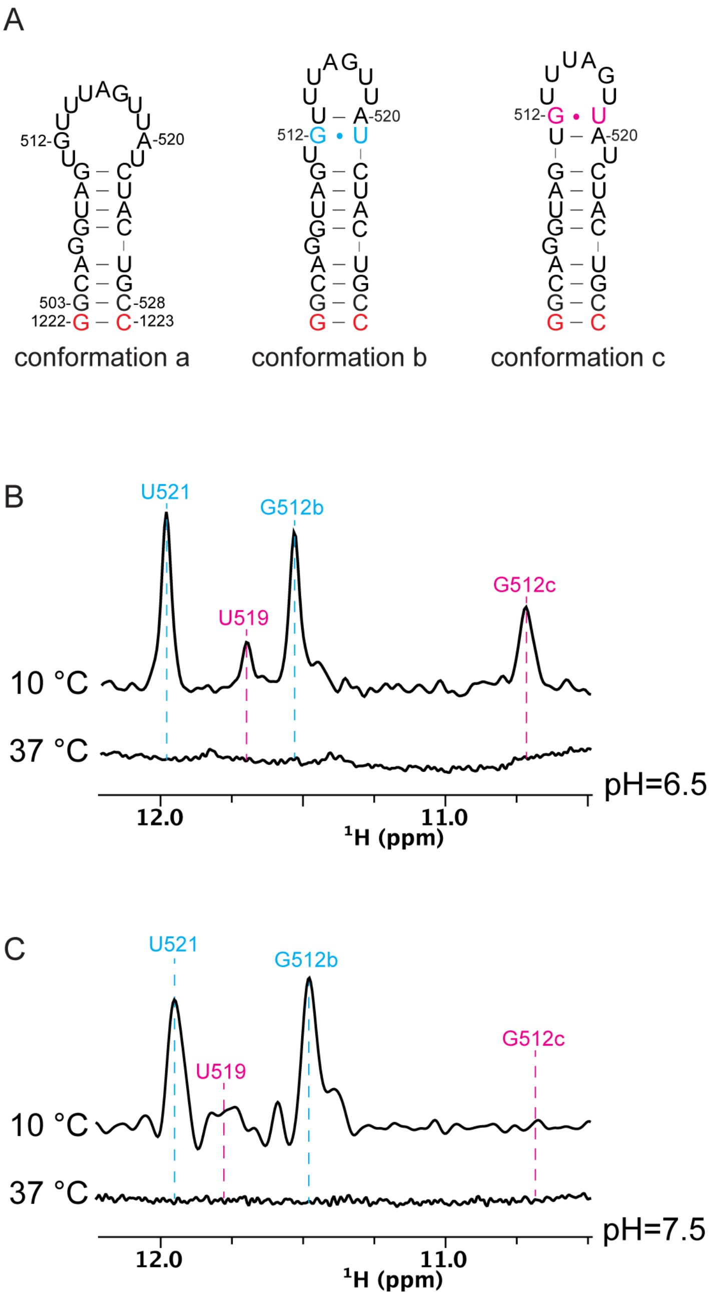

Figure S17. Temperature-dependence of 20a-frag4 imino proton resonances. (A) Secondary structures of alternative apical loop conformations. (B) Imino proton spectra of 20a-frag4 at pH 6.5. (C) Imino proton spectra of 20a-frag4 at pH 7.5. The NMR spectra were recorded at 0.5 mM RNA concentration, 50 mM K-phosphate buffer, 1 mM $MgCl_2$ and 90% H_2O/10% D_2O at 600 MHz and at the temperature and pH indicated within the figure.

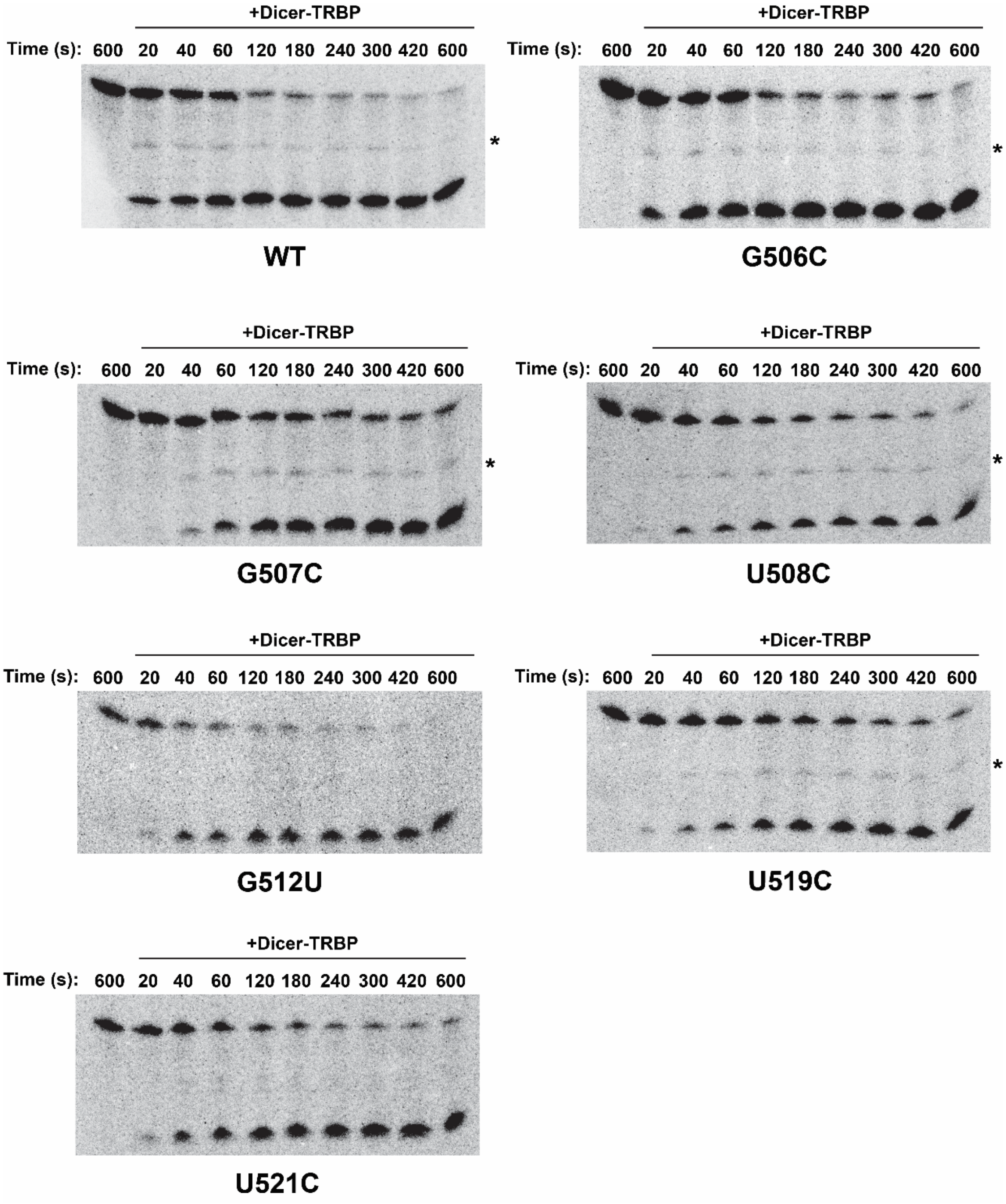

Figure S18. Representative gel images from *in vitro* Dicer-TRBP processing assays. *In vitro* Dicer-TRBP processing of ^{32}P-labeled pre-miR-20a WT and mutant RNAs. The asterisk (*) indicates a partially processed product which was included in the total RNA calculation when present.

Table S1. Chemical shift completeness.[a]

20a-frag1	A (% assigned)	C (% assigned)	G (% assigned)	U (% assigned)
H8/H6	100	100	100	100
H2/H5	100	100	/	100
H1′	100	100	100	100
H2′	100	100	100	100
H3′	91.7	100	100	100
C6/C8	100	100	100	100
C2	100	/	/	/
20a-frag2	A (% assigned)	C (% assigned)	G (% assigned)	U (% assigned)
H8/H6	100	100	100	100
H2/H5	100	100	/	100
H1′	100	100	100	100
H2′	100	100	100	100
H3′	100	100	100	100
C6/C8	100	100	100	100
C2	100	/	/	/
20a-frag3	A (% assigned)	C (% assigned)	G (% assigned)	U (% assigned)
H8/H6	100	100	100	100
H2/H5	100	100	/	100
H1′	100	100	100	100
H2′	100	100	100	100
H3′	100	100	100	100
C6/C8	100	100	100	100
C2	100	/	/	/
20a-frag4	A (% assigned)	C (% assigned)	G (% assigned)	U (% assigned)
H8/H6	100	100	100	100
H2/H5	100	100	/	100
H1′	100	100	100	100
H2′	100	100	100	100
H3′	100	100	100	100
C6/C8	100	100	100	100
C2	100	/	/	/
FL pre-miR-20a	A (% assigned)	C (% assigned)	G (% assigned)	U (% assigned)
H8/H6	100	100	100	100
H2/H5	100	100	/	100
H1′	100	100	100	100
H2′	100	100	100	100
H3′	100	100	100	100
C6/C8	100	100	100	100
C2	100	/	/	/

[a] "/" indicates a given atom is not present in the nucleoside.

Table S2. NMR restraints and structural statics for the FL pre-miR-20a structure.[a]

**CYANA**[b]	
NOE-derived restraints	390
Intraresidue	147
Sequential	205
Long-range (|i-j| > 1)	38
H-bond restraints	268
NOE restraints/residue	5.3
Target function (Å^2)	0.118 ± 0.0250
Upper distance viol. (Å^2)	0.00837 ± 0.000867
Lower distance viol. (Å^2)	0.00502 ± 0.000834
RMSD[c] (Å)	1.15 ± 0.344
**Amber**[d]	
Amber Energy	-16,532
Distance	4.688
Torsion	35.504
RMSD[c] (Å)	1.00 ± 0.308
**MolProbity analysis**[e]	
Clashscore	0.86
Probably wrong sugar pucker (%)	0
Bad backbone conformation (%)	6.5
Bad bonds (%)	0
Bad angles (%)	0

[a]Statistics are reported for the entire structure, unless otherwise indicated.
[b]Statistics for the 20 structures with lowest target function.
[c]Root mean square deviation. Statistics are reported for residues 481-510, 522-551
[d]Statistics for the 20 lowest energy structures.
[e]The 20 Amber-refined structures were evaluated using the MolProbity webserver.[1,2]

Table S3. SEC-SAXS data acquisition, sample details, data analysis, model fitting, and software used.

**(a) Sample details**	
Sample	FL pre-miR-20a
Organism	*Homo sapiens*
Sequence (5′ to 3′)	GGUAGCACUAAAGUGCUUAUAGUGCAGGUAGUGUUUAGUUAUCUACUGCAUUAUGA GCACUUAAAGUACUGCC
Extinction coefficient, ε_{260} (M^{-1} cm^{-1})	744800
M from chemical composition (kDa)	23.48
SEC-SAXS column	Superdex 75 10/300 Increase
Loading volume (μL)	260
Concentration (mg/mL)	1.3
Flow rate (mL/min)	0.6
Solvent	50 mM potassium phosphate buffer pH = 7.5, 1 mM $MgCl_2$, 50 mM NaCl, 5 mM BME
**(b) SAS data collection parameters**	
Instrument	BioCAT (beamline 18ID, APS) with a Dectris Eiger2 XE 9M detector
Wavelength (Å)	1.033
Beam size (μm)	30 × 150 (focused on detector)
Camera length (m)	3.663
q measurement range ($Å^{-1}$)	0.0029 – 0.42
Absolute scaling method	Glassy Carbon, NIST SRM 3600
Normalization	To transmitted intensity by beam-stop counter
Monitoring for radiation damage	Automated frame-by-frame comparison of relevant regions using CORMAP[3] implemented in BioXTAS RAW[4]
Exposure time	0.5 s exposure time with a 1 s total exposure period (0.5 s on, 0.5 s off) of entire SEC elution
Sample configuration	SEC-MALS-SAXS. Size separation used a Superdex 75 10/300 Increase column and a 1260 Infinity II HPLC (Agilent Technologies). UV data was measured in the Agilent, and MALS-DLS-RI data by DAWN HELEOS-II (17 MALS + 1 DLS channels) and Optilab T-rEX (RI) instruments (Wyatt Technology). SAXS data was measured in a sheath-flow cell,[5] effective path length 0.542 mm.
Sample temperature (°C)	22
**(c) Software employed for SAXS data reduction, analysis, and interpretation**	
Data reduction	$I(q)$ vs q and solvent subtraction using BioXTAS RAW 2.2.1[4]
Extinction coefficient estimate	Quest Calculate™ RNA Concentration Calculator *via* web server (https://www.aatbio.com/tools/calculate-RNA concentration)

Basic analyses, Guinier, $P(r)$, V_p	BioXTAS RAW 2.2.1[6] and GNOM from ATSAS 3.0.307/05/2025 13:52:00
Electron density modelling	DENSS[3]
Atomic structure modelling	FoXS[7,8] *via* web server (https://modbase.compbio.ucsf.edu/foxs/)
Three-dimensional graphic model representations	PyMOL (version 3.1.4.1)
**(d) Structural parameters**	
Guinier analysis	
$I(0)$ (cm^{-1})	0.01 ± 1.86×10^{-5}
R_g (Å)	30.57 ± 0.19
q_{min} (Å^{-1})	0.0029
qR_g max	1.001
Coefficient of correlation, R^2	0.936
M from V_c	25.2
$P(r)$ analysis	
$I(0)$ (cm^{-1})	0.01 ± 1.75×10^{-5}
R_g (Å)	31.29 ± 0.13
D_{max} (Å)	112
q-range (Å^{-1})	0.0029 – 0.4201
χ^2	0.872
**(e) Shape model-fitting results**	
DENSS (default parameters, 20 calculations)	
q-range for fitting	0.0029 – 0.4201
Symmetry, anisotropy assumptions	P1, none
Ambiguity score (AMBIMETER)[9]	2.155
χ^2 range	0.00283 - 0.06927
Model resolution (Å)	25.4 ± 3.9
**(f) Atomistic modeling**	
NMR structures	PDB entry 9OBM
FoXS	
χ^2	0.88
Predicted R_g (Å)	30.97
c_1, c_2	1.01, 2.97
**(g) SASBDB IDs for data and models**	
FL pre-miR-20a	SASXXXX

Table S4. Synthetic DNA templates and associated RNA constructs.

	5′-sequence-3′[a,b,c]	
Construct	DNA	RNA
20a-frag1	mGmGCAGTACTTTAAGTTCTCACTTTAGTGC TACC*TATAGTGAGTCGTATTA*	GGUAGCACUAAAGUGAGAACUUAA AGUACUGCC
20a-frag2	mGmGAAGTGCTCATAATTCTCACTATAAGC ACTTCC*TATAGTGAGTCGTATTA*	GGAAGUGCUUAUAGUGAGAAUUA UGAGCACUUCC
20a-frag3	mGmGTAATGCAGTAGTCTCCTACCTGCACT ACC*TATAGTGAGTCGTATTA*	GGUAGUGCAGGUAGGAGACUACUG CAUUACC
20a-frag4	mGmGCAGTAGATAACTAAACACTACCTGCC *TATAGGAGTCGTATTA*	GGCAGGUAGUGUUUAGUUAUCUAC UGCC

[a] m denotes 2′-*O*-Me modification of the primer.

[b] Italicized nucleotides correspond to the sequence complementary to the T7 promoter.

[c] Red nucleotides indicate non-native sequences.

Table S5. DNA primers for generation of the FL pre-miR-20a (NMR/SAXS) template.

	5′-sequence-3′[a]
Pre-miR-20a_F	TTCTAATACGACTCACTATAGGTAGCACTAAAGTGCTTATAGTGCAGGTAGTGT
Pre-miR-20a_R	mGmGCAGTACTTTAAGTGCTCATAATGCAGTAGATAACTAAACACTACCTGCA CTATAAGCA

[a] m denotes 2′-*O*-Me modification of the primer.

Table S6. DNA primers for HH-pre-miR-20a-HDV template.

	5′-sequence-3′
HH-20a-HDV_F	CCGGAATTCTAATACGACTCACTATAGGGCTCG
HH-20a-HDV_R	CCGTCGCGGATCCTAATGTGAGAATTGGCTACGTTGA

Table S7. Mutation DNA primers for processing constructs.

	5′-sequence-3′	Application
20a-G506C_F	TAGTGTTTAGTTATCTACTGCATTATG	Forward primer for G506C mutation
20a-G506C_R	CGTGCACTATAAGCACTTTAGACG	Reverse primer for G506C mutation
20a-G507C_F	AGTGTTTAGTTATCTACTGCATTATG	Forward primer for G507C mutation
20a-G507C_R	AGCTGCACTATAAGCACTTTAGAC	Reverse primer for G507C mutation
20a-U508C_F	ATAGTGCAGGCAGTGTTTAGTTATC	Forward primer for U508C mutation
20a-U508C_R	AAGCACTTTAGACGGTACCGG	Reverse primer for U508C mutation
20a-G512U_F	TGCAGGTAGTTTTTAGTTATCTAC	Forward primer for G512U mutation
20a-G512U_R	CTATAAGCACTTTAGACG	Reverse primer for G512U mutation
20a-U519C_F	AGTGTTTAGTCATCTACTGCATTATG	Forward primer for U519C mutation
20a-U519C_R	ACCTGCACTATAAGCACTTTAG	Reverse primer for U519C mutation
20a-U521C_F	TGTTTAGTTACCTACTGCATTATG	Forward primer for U521C mutation
20a-U521C_R	CTACCTGCACTATAAGCAC	Reverse primer for U521C mutation

Table S8. Amplification primers for template.

Amplification primers	5′-sequence-3′[a]	Application
UNIV-pUC19_E105	TCTTCGCTATTACGCCAGCTGGCGAAA	Forward primers for amplification of DNA templates for all pre-miR-20a processing constructs
HDV-AMP-R	mUmAATGTGAGAATTGGCTACGTTGA AACAACGCATTACCG	Reverse primers for amplification of DNA templates for all pre-miR-20a processing constructs

[a] m denotes 2′-*O*-Me modification of the primer.

Table S9. RNA sequence used for structural and processing studies.

Construct	5′-sequence-3′	Application
FL pre-miR-20a	GGUAGCACUAAAGUGCUUAUAGUGCAGGUAGUGUUU AGUUAUCUACUGCAUUAUGAGCACUUAAAGUACUGCC	NMR/SAXS studies
pre-miR-20a	UAAAGUGCUUAUAGUGCAGGUAGUGUUUAGUUAUCU ACUGCAUUAUGAGCACUUAAAG	Processing
pre-miR-20a_G506C	UAAAGUGCUUAUAGUGCACGUAGUGUUUAGUUAUCU ACUGCAUUAUGAGCACUUAAAG	Processing
pre-miR-20a_G507C	UAAAGUGCUUAUAGUGCAGCUAGUGUUUAGUUAUCU ACUGCAUUAUGAGCACUUAAAG	Processing
pre-miR-20a_U508C	UAAAGUGCUUAUAGUGCAGGCAGUGUUUAGUUAUCU ACUGCAUUAUGAGCACUUAAAG	Processing
pre-miR-20a_G512U	UAAAGUGCUUAUAGUGCAGGUAGUUUUUAGUUAUCU ACUGCAUUAUGAGCACUUAAAG	Processing
pre-miR-20a_U519C	UAAAGUGCUUAUAGUGCAGGUAGUGUUUAGUCAUCU ACUGCAUUAUGAGCACUUAAAG	Processing
pre-miR-20a_U521C	UAAAGUGCUUAUAGUGCAGGUAGUGUUUAGUUACCU ACUGCAUUAUGAGCACUUAAAG	Processing

Table S10. Dicer-TRBP processing of pre-miR-20a RNAs.[a]

RNA construct	% full cleavage (10 min.)	k_{obs} (s^{-1})	k_{obs} fold-change rel. to WT[b]	Adjusted p-value rel. to WT[c]	Significance level[c]
WT	97.0 ± 0.9	0.01581 ± 0.00101	----	----	----
G506C	97.1 ± 1.3	0.01450 ± 0.00029	0.92	0.2825	ns
G507C	83.6 ± 0.3	0.00893 ± 0.00107	0.56	<0.0001	****
U508C	88.2 ± 2.2	0.00484 ± 0.00047	0.31	<0.0001	****
G512U	98.0 ± 2.2	0.01838 ± 0.00075	1.16	0.0116	*
U519C	86.5 ± 0.7	0.00572 ± 0.00057	0.36	<0.0001	****
U521C	92.5 ± 1.6	0.01196 ± 0.00117	0.76	0.0004	***

[a]Values represent average and standard deviation from n = 2-3 independent assays.

[b]$k_{obs}(mut)/k_{obs}(WT)$

[c]Adjusted p-values are from one-way ANOVA with Dunnett's multiple comparison test (relative to mean of WT, $\alpha = 0.05$). * $p < 0.05$, ** $p < 0.01$, *** $p < 0.001$, **** $p < 0.0001$.

REFERENCES

1. Davis, I. W. *et al.* MolProbity: all-atom contacts and structure validation for proteins and nucleic acids. *Nucleic Acids Res* **35**, W375-83 (2007).
2. Chen, V. B. *et al.* MolProbity: all-atom structure validation for macromolecular crystallography. *Acta Crystallogr D Biol Crystallogr* **66**, 12–21 (2010).
3. Grant, T. D. Ab initio electron density determination directly from solution scattering data. *Nat Methods* **15**, 191–193 (2018).
4. Hopkins, J. B. BioXTAS RAW 2: new developments for a free open-source program for small-angle scattering data reduction and analysis. *J Appl Crystallogr* **57**, 194–208 (2024).
5. Kirby, N. *et al.* Improved radiation dose efficiency in solution SAXS using a sheath flow sample environment. *Acta Crystallogr D Struct Biol* **72**, 1254–1266 (2016).
6. Svergun, D. I. Determination of the regularization parameter in indirect-transform methods using perceptual criteria. *J Appl Crystallogr* **25**, 495–503 (1992).
7. Schneidman-Duhovny, D., Hammel, M., Tainer, J. A. & Sali, A. Accurate SAXS profile computation and its assessment by contrast variation experiments. *Biophys J* **105**, 962–974 (2013).
8. Schneidman-Duhovny, D., Hammel, M., Tainer, J. A. & Sali, A. FoXS, FoXSDock and MultiFoXS: Single-state and multi-state structural modeling of proteins and their complexes based on SAXS profiles. *Nucleic Acids Res* **44**, W424-429 (2016).
9. Petoukhov, M. V. & Svergun, D. I. Ambiguity assessment of small-angle scattering curves from monodisperse systems. *Acta Crystallogr D Biol Crystallogr* **71**, 1051–1058 (2015).
